# Expanding Access to Computed Tomographic Staging and Three-Dimensional Intensity Modulated Radiotherapy for Cervical Cancer in Ghana

**DOI:** 10.1200/GO.23.00266

**Published:** 2024-02-08

**Authors:** Aba Anoa Scott, Verna Vanderpuye, Mary-Ann Dadzie, Joel Yarney, Charles Akoto Aidoo, Judith Tackie, Stephen Kpatsi, Samuel Boateng, Tony Obeng-Mensah, Michael Nyamadi, Philip Odonkor, Tony Lam, Tony Tadic, Lian Velasco, Michael Milosevic

**Affiliations:** ^1^ Korle Bu Teaching Hospital, Accra, Ghana; ^2^ Radiation Medicine Program, Princess Margaret Cancer Centre, Toronto, ON, Canada; ^3^ Department of Radiation Oncology, University of Toronto, Toronto, ON, Canada; ^4^ Global Cancer Program, Princess Margaret Cancer Centre, Toronto, ON, Canada

## Abstract

**PURPOSE:**

To build capacity for improved treatment of locally advanced cervical cancer in Ghana, including computed tomography (CT) staging and intensity modulated radiotherapy (IMRT).

**MATERIALS AND METHODS:**

Patients with histologically confirmed cervical cancer were prospectively staged with abdominopelvic CT and ultrasound and offered the opportunity to have IMRT instead of conventional two-dimensional radiotherapy. The development of an efficient, high-quality, and safe IMRT program was facilitated by investment in new technology and comprehensive training of the interdisciplinary radiotherapy team in collaboration with a North American center of excellence.

**RESULTS:**

Of 215 patients with cervical cancer referred in 2022, 66% were able to afford CT scans and 26% were able to afford IMRT. Lymph node metastases were identified in 52% of patients by CT but in only 2% of patients by ultrasound. The use of CT resulted in 63% of patients being upstaged and changed treatment intent or radiation treatment volumes in 67% of patients. Patients who had IMRT experienced fewer acute side effects and were more likely to complete treatment as planned.

**CONCLUSION:**

It is feasible to provide state-of the-art cancer treatment with CT staging and IMRT to patients with cervical cancer in low-resource settings and achieve meaningful improvements in outcomes. It requires a broad commitment by program leadership to invest in technology and staff training. Major challenges include balancing improved clinical care with reduced patient throughput when radiation treatment capacity is constrained, and with the additional cost in the absence of universal health coverage.

## INTRODUCTION

Cervical cancer (CC) is the commonest gynecological cancer in Ghana and the second common cause of all cancer deaths.^1^ Although the majority of our patients present with locally advanced disease, they can still be cured with external-beam radiotherapy (EBRT) given concurrently with chemotherapy followed by brachytherapy, the current standard of care.^2^

**CONTEXT**

**Key Objective**
Describe a collaborative approach to implementing computed tomography (CT) staging and advanced intensity modulated radiotherapy (IMRT) for patients with cervical cancer in a low-resource setting.
**Knowledge Generated**
In this study, CT staging integrated with radiation treatment planning identified lymph node or other metastases in 58% of patients, changed the disease stage in 66% of patients, and altered management in 67% of patients. IMRT/three-dimensional (3D) radiotherapy was implemented without compromising overall treatment time or patient throughput. Among patients treated with IMRT/3D radiotherapy, there was less acute toxicity during treatment compared with two-dimensional radiotherapy leading to greater compliance and a higher likelihood of completing treatment as planned.
**Relevance**
The study provides a practical model for improving the management of cervical cancer in low resource settings and identifies important barriers for future attention, such as lack of public funding for cancer treatment.

The National Centre for Radiotherapy and Nuclear Medicine (NCRNM), Korle Bu Teaching Hospital (KBTH), Accra, Ghana, the largest oncology training and treatment center in the country, treats 200-250 patients with histologically confirmed CC annually. Until January 2022, almost all patients with CC were staged using simple chest x-rays (CXR) and abdominopelvic ultrasound. The importance of adequate imaging at diagnosis is acknowledged in the 2018 International Federation of Gynecology and Obstetrics staging classification, which previously was based on clinical criteria alone.^3^ Computed tomography (CT) scans, positron emission tomography-CT scans, and magnetic resonance scans are frequently used for CC staging in high-income countries,^4^ although not routinely in many low-resource settings such as Ghana^5-7^ because of availability and cost. In Ghana, there are a total of 46 CT scanners serving a population of 31 million, with a mean scanner-to-population ratio of 1.46/million people.^8^ However, CT imaging is increasingly being incorporated into EBRT planning in low-resource countries, providing an opportunity to obtain valuable information about the local extent of disease and lymph node and distant metastases^7^ not previously available from clinical examination or ultrasound.

Patients with CC who presented to the NCRNM were traditionally treated using two-dimensional (2D) EBRT techniques. These techniques are simple and allow rapid treatment of a large number of patients but are associated with high toxicities. Three-dimensional (3D), intensity modulated radiotherapy (IMRT) is now the standard of practice in high-income countries and has been shown to improve tumor control and reduce side effects.^9-12^ A recent phase III clinical trial in gynecological cancer reported better patient-reported quality of life with IMRT compared with 2D EBRT.^12^

Two previous studies conducted at NCRNM reported poorer treatment outcomes for patients with CC compared with data from high-income countries.^13,14^ In both studies, patients were staged using CXR and abdominopelvic ultrasound and treated using 2D techniques. With this background, and with the objective of becoming a center of excellence for radiotherapy in Ghana and a hub for education and knowledge translation in sub-Saharan Africa (SSA), NCRNM made significant investment in technology and training to migrate patients with cervical and other cancers from 2D to 3D EBRT. This included the installation of a CT simulator and a six-megavolt (MV) linear accelerator with IMRT capability in 2019 after many years of using a conventional simulator and ^60^Cobalt teletherapy machine, and a ^60^Cobalt high-dose-rate brachytherapy and treatment system with 3D capability.

This report describes the advances at NCRNM in a 1-year period from January 2022 to December 2022 to incorporate CT staging into the management of patients with CC and migrate treatment from 2D to 3D/IMRT-based EBRT.

## MATERIALS AND METHODS

This study was conducted at the NCRNM. Institutional review board approval was obtained from the KBTH. The study was open to all patients with a histologic diagnosis of CC who presented to the NCRNM with ECOG performance status 0-2.

### Training and Support

A radiation oncologist (A.A.S.) from NCRNM completed a 1-year clinical fellowship at the Princess Margaret Cancer Centre (PMH) in Toronto, Canada, to develop competencies in modern 3D radiotherapy techniques for CC and other gynecological cancers. To further accelerate the migration from 2D to 3D treatment, physicists at NCRNM received online IMRT training from the team at PMH. We built on and adapted some of the procedures in place at PMH to reflect the practice environment in Ghana, for example, a nomenclature template for regions of interest to facilitate contouring and treatment planning using the systems in place at NCRNM.

The training curriculum for radiation oncologists, physicists, and radiotherapists at NCRNM had been revised in recent years to include IMRT. In addition, the linear accelerator manufacturer provided training to strengthen IMRT plan delivery and quality assurance to practicing radiotherapists. These initiatives helped to build frontline capacity for efficient and safe IMRT at NCRNM before the first patient on the study being treated.

### CT Imaging for Staging and EBRT Planning

Before this study, all patients were staged with CXR and abdominopelvic ultrasound. All patients presenting after the study opened were encouraged to have CT imaging of the abdomen and pelvis. There is no insurance coverage for CC treatment in Ghana and patients pay out of pocket. Hence, not all patients were able to afford a CT scan.

CT scans were obtained in treatment position using the CT simulator with and without intravenous contrast. Patients with glomerular filtration rate <30 mL/min/1.73 m^2^ did not receive contrast. The scans provided previously unavailable information about the extent of local disease, lymph node metastases, and distant metastases to inform stage assignment and treatment recommendations. When curative radiotherapy was recommended, the scans also provided the imaging data sets required for IMRT planning. Patients were asked to empty their bladder, drink 500 mL of water, and wait for 30 minutes before imaging. CT scans were read by radiologists with expertise in abdominopelvic imaging. A lymph node was considered positive if it measured 1 cm or more in the short axis and had central necrosis or rim-enhancing features.^15^

### IMRT Planning and Delivery

Radiation Therapy Oncology Group (RTOG) guidelines for target and organs at risk (OARs) delineation were adapted to our clinical setting.^16^ The primary clinical target volume (CTVp) included the gross tumor, the cervix, 2 cm of uninvolved vagina caudal to the primary tumor, the entire uterus, and the parametrium. The primary internal target volumes (ITVp) were generated using a standard 1-cm expansion of the CTVp in the cranial, caudal, anterior, and posterior directions, and a 5-mm expansion in the lateral directions, edited to respect anatomic barriers defined by muscle and bone.^17,18^

For those without enlarged lymph nodes on CT, the lymph node clinical target volume (CTVn) was defined as the obturator, internal, and external iliac to the level of the common iliac bifurcation. For those with enlarged internal/external iliac nodes, the CTVn was expanded to also include the common iliac chain to the level of the aortic bifurcation. For those with enlarged common iliac or low para-aortic lymph nodes below the renal hilum, the CTVn was expanded further to encompass the para-aortic chain to the level of T12/L1. Patients with bulky para-aortic nodes >3 cm or enlarged lymph nodes cephalad to the renal hilum were treated with palliative intention.

Isotropic 7-mm expansions on the ITVp and CTVn were used to generate the primary and nodal planning target volumes, adapting the 5-mm margins used at PMH to compensate for the lack of once a day kV imaging at NCRNM.

**FIG 1:**
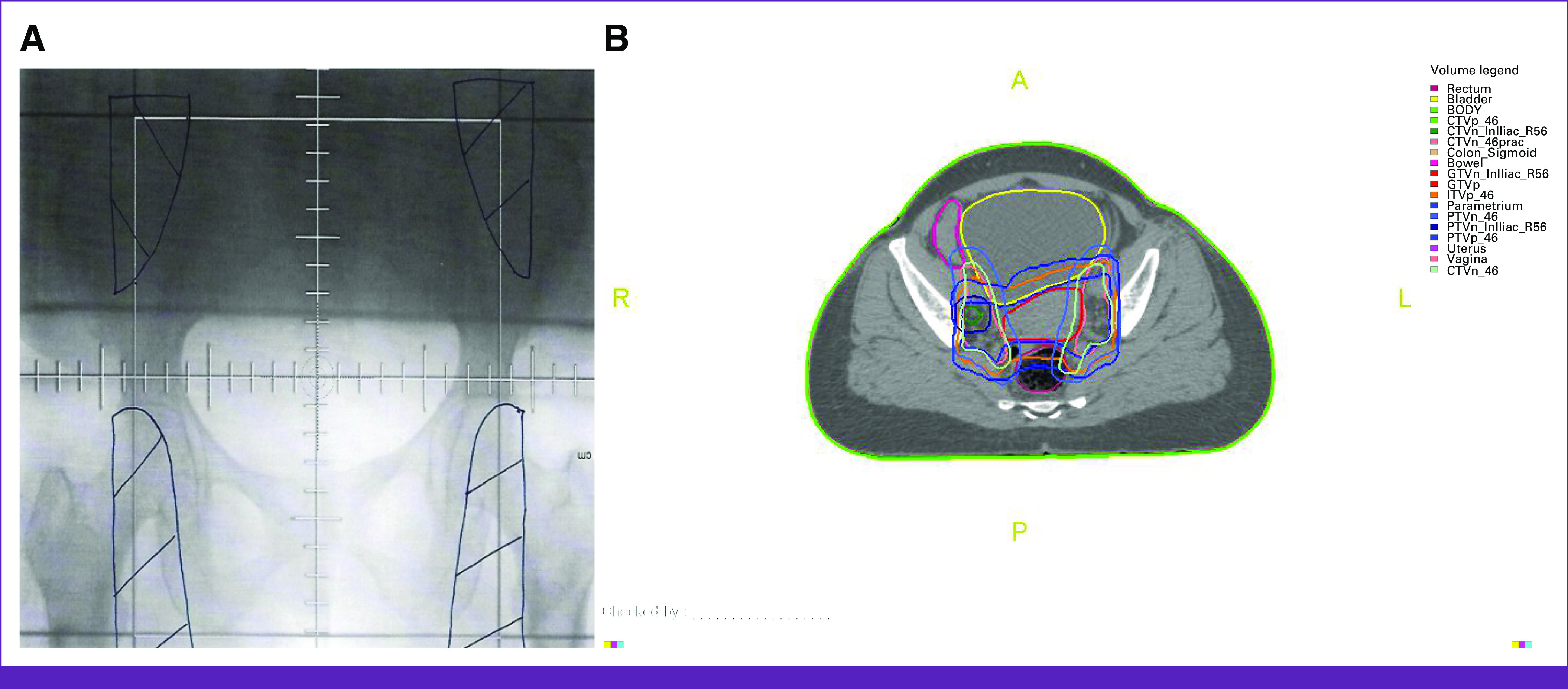
Radiotherapy planning for two representative patients with cervical cancer, (A) one treated with conventional 2D EBRT and (B) the second with IMRT. The target and OAR contours for the IMRT plan are shown in (B). 2D, two-dimensional; CTVn, node clinical target volume; CTVp, primary clinical target volume; EBRT, external-beam radiotherapy; GTVn, nodal gross tumor volume; GTVp, primary gross tumor volume; IMRT, intensity modulated radiotherapy; ITVp, primary internal target volume; OAR, organs at risk; PTVn, nodal planning target volume; PTVp, primary planning target volume.

Contoured OARs included the bladder, small bowel, sigmoid, and rectum, as well as the kidneys, duodenum, and spinal cord when the para-aortic lymph nodes were treated. Figure 1 shows the target and OAR contours in a typical patient with CC.

**FIG 2:**
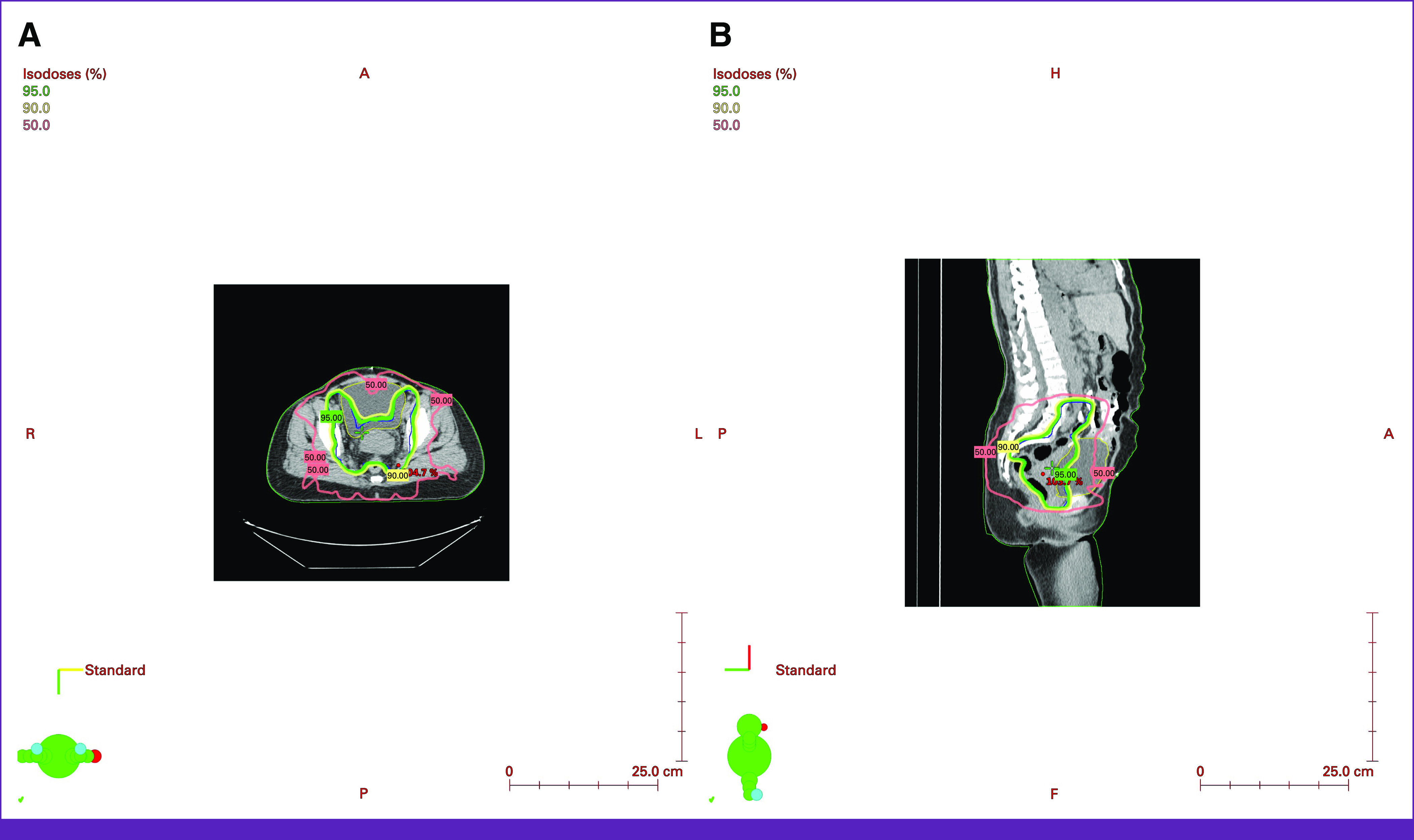
IMRT plan for a representative patient with cervical cancer showing (A) axial and (B) mid-sagittal images. The 95% (green), 90% (yellow), and 50% (red) isodose lines are indicated. IMRT, intensity modulated radiotherapy.

IMRT treatment plans with seven coplanar six MV beams were generated (Eclipse, version 13.6, Varian Medical Systems, Palo Alto, CA) by the team in Ghana using target and OAR dose constraints adapted from PMH and the RTOG 1203 protocol. Figure 2 shows a representative IMRT plan.

The dose prescription was 46 Gy in 23 once a day fractions. This was followed by a sequential IMRT boost to involved lymph nodes, commonly 10 Gy in five once a day fractions, but modified in individual patients on the basis of adjacent normal tissues. The contours and treatment plans for the initial patients were peer-reviewed by the NCRNM and PMH teams together, bearing in mind the available resources in Ghana. Peer review was done via virtual coaching sessions to facilitate discussion and learning. Once the technique was established, peer review of all subsequent IMRT plans was done by the NCRNM team alone.

The radiation treatment machine at NCRNM does not have an onboard cone beam CT or kV imager. Therefore, treatment verification was done once per week using orthogonal images produced with the MV beam. The MV images were superimposed onto the planning CT images using clearly delineated anatomic bony landmarks, ensuring reproducible daily setup.

### Conventional 2D EBRT

For patients treated using 2D techniques, a conventional simulator was used to delineate the treatment fields using bony landmarks as guides. Generally, an anterior-posterior (AP/PA) opposed ^60^Co fields (Fig 1) were used. Lateral fields were added only if the patient had a separation of >21 cm to reduce toxicity to bowel. The prescribed dose was 46 Gy in 23 once a day fractions. Patients with parametrial or pelvis side wall involvement received a boost of 10-12 Gy in 2 Gy once a day fractions using the same AP/PA fields with the cephalad border reduced to the sacro-iliac (SI) joint, and a 4-cm wide midline shield was added to reduce the risk of bowel, bladder, and rectal toxicity.

All patients treated with curative intention received concurrent once a week cisplatin at a dose of 40 mg/m^2^ unless contraindicated.

## RESULTS

**FIG 3:**
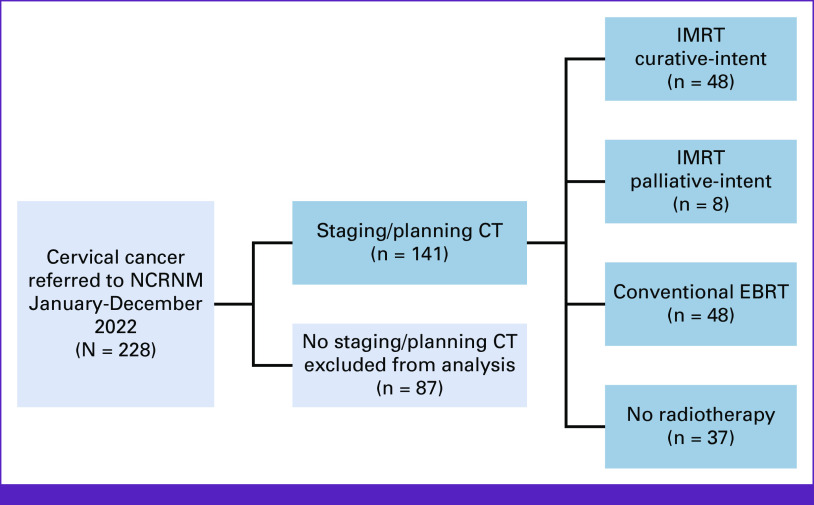
CONSORT diagram for the patients with cervical cancer referred to NCRNM from January to December 2022 inclusive. The patients included in the analysis are represented by the blue boxes. CT, computed tomography; EBRT, external-beam radiotherapy; IMRT, intensity modulated radiotherapy; NCRNM, National Centre for Radiotherapy and Nuclear Medicine.

Figure 3 is the CONSORT diagram for patients with CC referred to NCRNM and included in the analysis. Over the study period, 228 patients with CC presented to NCRNM. Thirteen patients defaulted workup and were excluded. The remaining 215 patients were offered the opportunity to participate in this study of abdominopelvic CT scanning for staging and radiotherapy planning. A total of 141 patients (66%) were able to afford CT scans and agreed to participate. These patients also had a CXR or CT chest for staging and some had an initial ultrasound to evaluate for liver metastases and hydronephrosis. They formed the analysis cohort. Development of IMRT at NCRNM took approximately 4 months, after which it was routinely made available to patients. Of the 141 patients who had CT scans, 56 (40%) were also able to afford IMRT/3D. The others received conventional EBRT (34%) or no radiotherapy at all (26%). Baseline patient and tumor characteristics are shown in Table 1.

**TABLE 1:** Baseline Patient and Tumor Characteristics

Patient or Tumor Characteristic	Cohort (n = 141)
Age, years	
Median (range)	58 (28-87)
Histologic type, No. (%)	
Squamous cell carcinoma	125 (88.7)
Adenocarcinoma	12 (8.5)
Adenosquamous carcinoma	2 (1.4)
Others	2 (1.4)
FIGO 2018 stage, No. (%)	
IB	5 (3.5)
IIA	6 (4.3)
IIB	17 (12.1)
IIIB	18 (12.8)
IIIC	64 (45.4)
IVA	8 (5.7)
IVB	23 (16.3)
ECOG performance status, No. (%)	
0	95 (67.4)
1	25 (17.7)
2	21 (14.9)
Imaging, No.	
CT and ultrasound	91
CT alone	50
Metastasis, No.	
Lymph node	82
Other	64
EBRT, No.	
IMRT/3D	56
Conventional EBRT	48
None	37

Abbreviations: 3D, three-dimensional; CT, computed tomography; EBRT, external-beam radiotherapy; ECOG, Eastern Clinical Oncology Group; FIGO, International Federation of Gynecology and Obstetrics; IMRT, intensity modulated radiotherapy.

Of the 141 patients, the most common finding on abdominopelvic CT was lymph node metastases in 82 patients (58%). Comparing the 91 patients who had both CT and ultrasound (Table 2), 47 (52%) had lymph node metastases diagnosed by CT. CT identified new sites of disease and/or altered management in 61 of the 91 patients (67%). As summarized in Table 3, the use of CT resulted in 57 patients (63%) being upstaged, most frequently because of lymph node metastases (n = 37), distant metastases (n = 12), or rectal or bladder infiltration (n = 4) not recognized by clinical examination or ultrasound. Three patients (5%) were downstaged.

**TABLE 2:** Comparison of Abdominal/Pelvic CT Versus Abdominal/Pelvic Ultrasound Detection of Metastases in 91 Patients Who Had Both Imaging Modalities

Abnormal Finding on Imaging	Detected by Abdominal/Pelvic CT, No. (%)	Detected by Abdominal/Pelvic Ultrasound, No. (%)
Pelvic ± para-aortic lymph node metastases	47 (52)	2 (2)
Hydronephrosis	9 (10)	6 (7)
Liver metastasis	5 (5)	0
Incidental abdominal/pelvic findings	5 (5)	0

Abbreviation: CT, computed tomography.

**TABLE 3:** Change in Stage With the Addition of Abdominopelvic CT in 91 Patients Who Had Both Imaging Modalities

2009 FIGO Stage	2018 FIGO Stage	No. of Patients
IB	IIA	1
IB	IIIC	5
IIA	IIB	1
IIA	IIIC	5
IIB	IIIB	2
IIB	IIIC	10
IIB	IVA	3
IIB	IVB	3
IIIA	IIIC	3
IIIA	IVB	4
IIIB	IIIC	14
IIIB	IVA	1
IIIB	IVB	5
IVA	IIIC	2
IVB	IIIB	1
Total		60

Abbreviations: CT, computed tomography; FIGO, International Federation of Gynecology and Obstetrics.

Fifty-six patients were treated with IMRT/3D plans, of whom 48 were treated with curative intent, and all but one completed treatment as planned. The remaining eight were treated with palliative intent. Among the 48 patients who received conventional EBRT, 20 were treated with curative intent and completed treatment as planned, nine began curative-intent treatment but did not complete, and 17 were treated with palliative intent. The median cumulative dose from EBRT and brachytherapy for those treated with curative intent was 86 Gy (range, 80-90 Gy). IMRT required 3-4 hours per patient for contouring and planning versus <0.5 hours for a conventional 2D EBRT plan. IMRT delivery times on the treatment unit were also longer because of the larger number of fields treated per patient. For patients treated with curative intent, the median treatment duration including brachytherapy was 54 days (range, 50-56 days) and 54 days (range, 51-54 days) for the 3D/IMRT and 2D cohorts, respectively. The median number of once a week cisplatin doses was five (range, 1-6) for patients in the IMRT cohort and five (range, 3-5) for patients in the 2D cohort. Patients treated with palliative intent received doses ranging from 10 Gy in a single fraction to 30 Gy in 10 once a day fractions.

Of those treated, 39 patients (38%) experienced acute toxicity during or within 6 weeks of completing radiotherapy, measured using Common Terminology Criteria for Adverse Events version 5.^19^ Thirty patients (29%) experienced grade 1 or 2 toxicity, most commonly skin desquamation and/or diarrhea. Fifteen patients (14%) experienced grade 3 toxicity, including skin desquamation, diarrhea, and/or cystitis. There was no grade 4 toxicity. Eighteen patients (17%) experienced overlapping toxicities, with 50% having at least one grade 3 toxicity. There was a pattern of less acute toxicity of any grade in patients receiving IMRT than in those receiving conventional treatment (diarrhea 8% *v* 45%, dysuria 14% *v* 29%, skin desquamation 14% *v* 29%, respectively). Patient follow-up was too short to meaningfully compare local tumor control, patient survival, or late toxicity between IMRT versus conventional EBRT.

## DISCUSSION

CC is a major health problem in many African countries and will remain so for years despite improvements in early detection and vaccination.^20^ The majority of patients present with locally advanced disease can be cured with EBRT and concurrent cisplatin followed by brachytherapy. However, radiotherapy continues to be unavailable or severely capacity limited in many low-resource countries where CC is endemic and restricted to the use of older treatment techniques that are associated with high recurrence rates and toxicity.^21,22^

This report outlines a staged approach to implementing advanced radiotherapy for CC at NCRNM, beginning with investment in a state-of-the-art CT simulator and IMRT-capable treatment machine followed by comprehensive training of a radiation oncologist in gynecological cancer radiation oncology at the PMH and continued collaboration between the NCRNM and PMH interdisciplinary teams afterward. It demonstrates the feasibility of using modern radiotherapy in a low-resource setting and provides early evidence of improved clinical outcomes compared with conventional treatment, specifically fewer acute side effects leading to improved treatment compliance. The study also highlights some of the challenges faced in low-resource countries, such as the financial toxicity of cancer treatment in the absence of public health insurance.

Accurate staging of patients with CC is important for determining prognosis, selecting appropriate treatment, and defining appropriate target volumes. Before this study opened, the proportion of patients with CC at NCRNM with lymph node metastases was unknown. We capitalized on the availability of a CT simulator to study CT staging of the abdomen and pelvis compared with our traditional approach with ultrasound. The early results were very encouraging (16 of the first 21 patients had lymph node metastases not seen on ultrasound) and helped to build broad support for CT staging across the program. In the final analysis, CT identified lymph node or other metastases in 58% of patients, changed the disease stage in 66% of patients, and altered management (curative *v* palliative intent, modified radiation treatment volume or dose) in 67% of patients. Incomplete staging may in part explain the worse stage-for-stage survival of patients with CC previously treated at NCRNM relative to patients treated at a cancer center in the United States.^13,14^

IMRT plans can be sculpted to conform to the cancer target while protecting normal tissues. Early results from this study suggest less acute toxicity during treatment compared with our previous approach, consistent with other reports.^10-12^ Patients who received IMRT were more likely to complete treatment as planned, in part because it was better tolerated. The follow-up of patients in the study was too short to comment meaningfully on tumor control, patient survival, or late toxicity. Of note, online treatment verification of patients in this study was done using once a week orthogonal images produced with the MV treatment beam. Soft tissue tumor and normal tissue motion may have contributed to residual targeting errors, offsetting some of the benefit of IMRT. This highlights the importance of equipping radiation treatment equipment in Ghana and other low-resource countries with more advanced cone beam CT imagers and better understanding of how often online imaging is required during IMRT (from once a week to once a day), balancing targeting accuracy against patient throughput and the available resources.

Although generating and delivering IMRT was more tasking and required more resources, it allowed our team at NCRNM to treat women with CCs in a best-practice manner. While there is the potential for IMRT to take longer, extend overall treatment time and reduce capacity so that fewer patients can be treated, we fine-tuned our processes to minimize these effects and saw no adverse consequences. The impact of this investment in technology and program development for CC should translate to other cancers and contribute broadly to establishing NCRNM as a center of excellence for radiotherapy in Ghana and a hub for education and knowledge translation in SSA.

Brachytherapy is a critical component of curative radiotherapy for locally advanced CC.^23^ Image-based 3D brachytherapy has largely replaced 2D brachytherapy, becoming the standard of care.^24^ Although beyond the scope of this report, CT-guided brachytherapy is currently being developed at NCRNM to further enhance tumor control and reduce toxicity.

An important limitation of the study is patient selection on the basis of the ability to pay for CT scans and IMRT. Although significant strides were made by NCRNM to make CT staging and IMRT available, access remains limited because the cost of CC treatment is not covered by the National Health Insurance Scheme of Ghana. The cost of CT was approximately $100 in US dollars (USD) in 2022. The cost of a full course of IMRT was considerably higher at approximately $1000 USD and affordable by only 26% of patients overall. A second important limitation is short patient follow-up. The focus of the manuscript was to describe the development of a program to expand access to 3D/IMRT for patients with CC in Ghana, not to comprehensively report clinical outcomes. We found less acute toxicity during treatment with IMRT that resulted in improved treatment compliance. However, a more thorough analysis will require longer patient follow-up and a more systematic approach to collecting long-term outcome data.

In conclusion, the accomplishments at NCRNM within a short period of time demonstrate that it is feasible to provide state-of the-art cancer treatment with CT staging and 3D conformal radiotherapy to patients with CC in low-resource settings. It requires a broad commitment by program leadership to invest in technology and staff training and, in our case, a sustained collaboration with a center of excellence to assist with program development. Major challenges include balancing improved clinical care with reduced patient throughput when radiation treatment capacity is constrained and with the additional cost when cancer treatment is paid out of pocket.

## SUPPORT

Supported by grants awarded to A.A.S. by the ASCO Conquer Cancer Foundation and the Princess Margaret Cancer Centre Global Cancer Program.
